# Organic and Inorganic Pollutants, Oxidative Stress Biomarkers, and Electrophoretic Protein Profiles in an Endangered Thresher Shark (*Alopias vulpinus*) From Southeastern Brazil: A Case Study

**DOI:** 10.1002/tox.70026

**Published:** 2026-01-15

**Authors:** Sidney Fernandes Sales Junior, Amanda Pontes Lopes, Heloíse Martins de Souza, Regina Fonseca, Enrico Mendes Saggioro, Francielli Monteiro Casanova, Kamila Cezar Gramlich, Tatiana Dillenburg Saint'Pierre, Carlos German Massone, Renato Carreira, Rachel Ann Hauser‐Davis

**Affiliations:** ^1^ Laboratório de Avaliação e Promoção da Saúde Ambiental (LAPSA) Instituto Oswaldo Cruz, Fundação Oswaldo Cruz (Fiocruz) Rio de Janeiro RJ Brazil; ^2^ Programa de Pós‐Graduação em Biodiversidade e Saúde Instituto Oswaldo Cruz, Fundação Oswaldo Cruz (Fiocruz) Rio de Janeiro RJ Brazil; ^3^ Laboratório de Espectrometria Atômica (LABSPECTRO), Departamento de Química Pontifícia Universidade Católica do Rio de Janeiro (PUC‐Rio) Rio de Janeiro RJ Brazil; ^4^ Laboratório de Estudos Marinhos e Ambientais (LABMAM), Departamento de Química Pontifícia Universidade Católica do Rio de Janeiro (PUC‐Rio) Rio de Janeiro RJ Brazil

**Keywords:** Conservation, Elasmobranch, Metal(loids), Multiclass contaminant screening, Oxidative stress, POPs, SDS–PAGE

## Abstract

A comprehensive screening of different environmental contaminants (total metals, thermostable metal fractions associated with detoxification, and persistent organic pollutants) was conducted in an endangered common thresher shark ( *Alopias vulpinus* ) individual incidentally captured in southeastern Brazil. Stress biomarkers were assessed to evaluate physiological responses, and SDS–PAGE was applied to identify thermostable protein profiles. Most metals were more abundant in total than in thermostable fractions, suggesting partial cellular absorption, likely through compartmentalization in organelles or binding to metallothioneins. Of 25 screened pesticides, only p,p′‐DDT and Mirex were detected, with unexpectedly high levels observed in liver. Several low molecular weight PAHs were also found in both tissues, especially muscle, suggesting chronic exposure and bioaccumulation. The SDS–PAGE revealed ~15 kDa bands, consistent with metallothioneins, and higher bands possibly corresponding to matrix metalloproteinases. Poor resolution in the Ampullae of Lorenzini was likely due to high salt content. Antioxidant biomarkers showed tissue‐specific patterns, with high H_2_O_2_ levels and SOD activity in gills, blood, and brain, suggesting oxidative stress. Further studies are, however, required, as a sample number of one precludes broad conclusions at the species level. Despite this limitation, the study provides valuable preliminary insights and a baseline for *Alopias* spp. The integrated biochemical and molecular approach applied herein may aid in detecting early physiological stress and sublethal contamination effects and, combined with ecological and life‐history data, can inform conservation strategies, such as habitat prioritization, pollutant mitigation, and monitoring programs, to support the long‐term survival and population viability of this and other vulnerable shark species.

## 1 Introduction

Chemical contamination has become ubiquitous throughout the world's oceans, with significant negative physiological effects verified in many trophic level representatives [1, 2, 3, 4, 5]. This is especially true for top predators, due to the bioaccumulation and biomagnification processes that take place for many pollutants [6, 7, 8]. This is the case for many shark species, which, besides being mostly top predators, also exhibit certain life characteristics, such as longevity, slow growth and low fecundity, that make them highly vulnerable to chemical contamination [9, 10, 11]. However, only recently has chemical pollution been deemed of significant concern for this taxonomic group [12], currently ranked as one of the five main causes for the high population declines observed in the last decades for these animals [82]. Because of this, ecotoxicological evaluations concerning sharks are still not as advanced as they should be, hindering adequate conservation efforts, with many contaminants and their physiological effects still understudied in these organisms [13, 14].

Many elasmobranch stocks have declined severely in the last decades [15], including thresher sharks, namely *Alopias pelagicus* [16], *Alopias superciliosus* [17], and *Alopias vulpinus* [18, 19]. The Common or Atlantic thresher shark ( *A. vulpinus* ) is pelagic with oceanic‐coastal habits in tropical, subtropical, and temperate regions, and a cosmopolitan occurrence [20]. This species preys mainly on schools of fish and squid [21, 22], using their tail to strike and stun their prey [22, 23]. It is an aplacental viviparous oophagous species [24], with litters ranging from two to six pups [25]. The International Union for Conservation of Nature currently categorizes *A. vulpinus* as vulnerable, with a decreasing population trend, severely fragmented populations and a continuing decline of mature individuals [25]. The species global population reduction has been recently estimated at 30%–49% over the last three generations, with no action recovery plans or systematic monitoring schemes in place [25].

This case study aims to report a baseline screening of organic and inorganic contaminants, oxidative stress biomarkers and electrophoretic protein profiles in an *A. vulpinus* individual incidentally captured in Rio de Janeiro, southeastern Brazil, by artisanal fishery activities. In this sense, ecotoxicological evaluations applied to wildlife assessments are important to investigate, protect, and manage wildlife conservation in response to anthropogenic contamination [26].

## 2 Methodology

### 2.1 *Alopias vulpinus* Sampling and Processing

**FIGURE 1:**
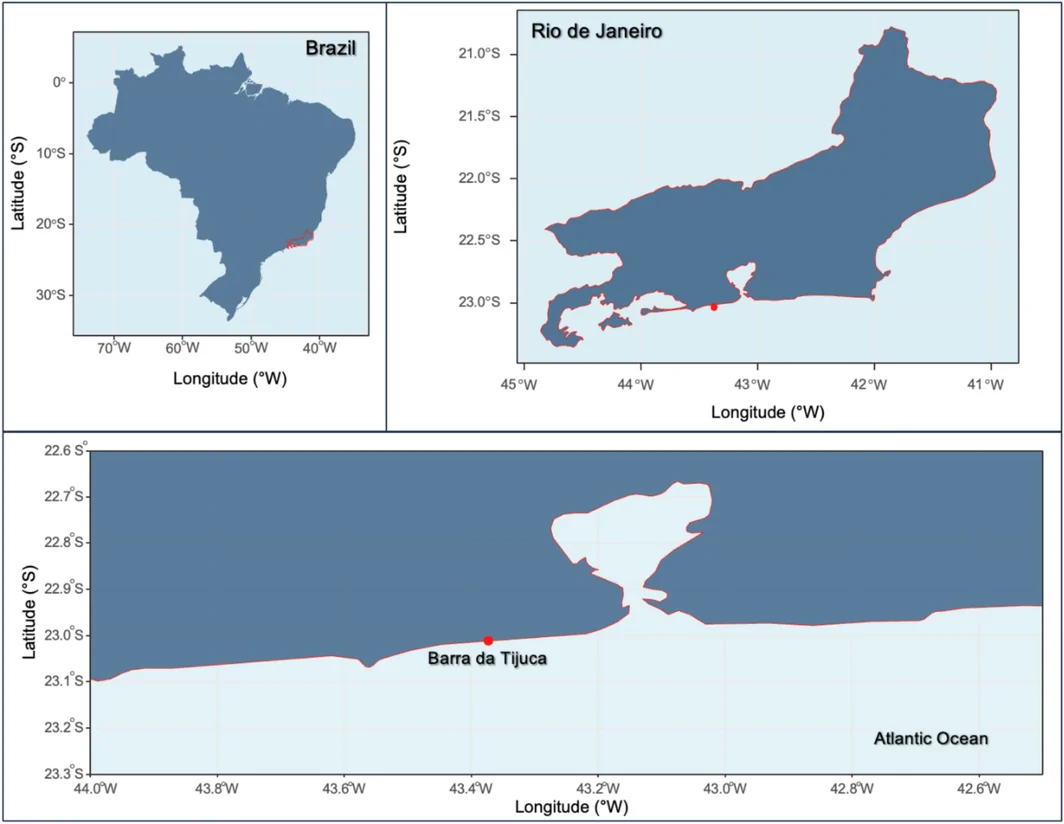
Map of the *Alopias vulpinus* sampling location in Barra da Tijuca, Rio de Janeiro, southeastern Brazil.

The *A. vulpinus* specimen was incidentally caught by artisanal fishers at Barra da Tijuca, in the metropolitan region of the city of Rio de Janeiro, southeastern Brazil, who fish with gill nets at about 200 m from the shore (Figure 1).

Following transportation to the laboratory, the individual was confirmed as juvenile female *A. vulpinus* based on total length (TL), where females mature at 260–465 cm TL [27, 28], and on diagnostic features described in [29] sexed, weighed, measured, and dissected according to standard US Environmental Protection Agency (EPA) protocol EPA/600/R‐92/111 [30]. The ampullae of Lorenzini, eyes, bile, fin, muscle, brain, rectal gland, pancreas, gills, heart, spleen, uterus, ovary, kidney, liver, and blood were removed for further processing.

### 2.2 Biomorphometric Calculations

The condition factor (CF) of the thresher shark was calculated as CF = 100 x (TW/TL^3^), where TW = total fish weight (g) and TL = total fish length (cm); the hepatosomatic index (HSI), calculated as HSI = 100 x (LW/TW), where LW = liver weight (g) and TW = total fish weight (cm); and the gonadosomatic Index (GSI), calculated as GSI = 100 x (GW/TW), where LW = gonad weight (g) and TW = total fish weight (cm).

### 2.3 Eletrophoretic Thermostable Protein Profile Characterization

Eletrophoretic protein characterizations of the shark organs and biological fluid were carried out by SDS–PAGE electrophoresis using the thermostable protein fraction obtained as detailed in the oxidative stress methodology section for metallothionein (MT) extraction. Total protein contents of each sample were quantified by the Lowry method, modified by Peterson using bovine serum albumin (BSA) as standard [31]. A total of 35 μg were applied to each gel lane. Electrophoretic runs were carried out on 15% sodium dodecyl sulfate polyacrylamide gels (SDS–PAGE) at 175 V and 0.3 A, according to Laemmli [32]. Gels were stained by silver staining as described by Heukeshoven and Dernick [33], with minor modifications. The molecular weights of the protein bands were determined using Biorad's Precision Plus ProteinTM Dual Color Standards.

### 2.4 Metal and Metalloid Determinations

Metal and metalloids were determined by inductively coupled plasma mass spectroscopy, ICP‐MS NexIon 300X (Perkin Elmer‐Sciex, Norwalk, CT, USA). Briefly, the samples were decomposed for 12 h in an acid medium (HNO_3_ 67% v/v/), heated at 100°C for 5 h in capped Falcon‐type 15 mL tubes, to avoid loss of volatile elements [34] and made‐up with ultrapure water (resistivity > 18.0 MΩ cm), for the ICP‐MS analysis. These contaminants were determined in both crude organ/fluid samples and in the thermostable purified fractions following MT extraction as detailed in the oxidative stress biomarkers section, to assess MT‐bound metals and metalloids. Multielemental external calibration was conducted using a mixed Merck IV standard solution and Rh was introduced online from a 20 mg L^−1^ solution as the internal standard. All sample determinations were performed in triplicate. Analytical curve correlation coefficients were always above 0.995. The method Limit of Quantification (LOQ) for each investigated element was determined following Brazilian National Institute of Metrology, Quality, and Technology standards [35], calculated as LOQ = (10 × SD × df)/slope of the calibration curve, where SD represents the standard deviation of the ratio between the analytical signal and the internal standard signal of 10 blanks, and df is the sample dilution factor (Table S1). The observed and certified values for the certified reference materials (BCR‐668—mussel tissue, DORM‐2—fish tissue, and ERMBB422—fish tissue, both from the European Commission) and recovery percentages ranged from 70% to 120%, satisfactory as per Eurachem standards [36].

### 2.5 Persistent Organic Pollutant Determinations

Concerning organic compounds, both PAHs and pesticides were determined. Several PAHs were investigated, encompassing naphthalene, acenaphthylene, acenaphthene, fluorene, dibenzothiophene, phenanthrene, anthracene, fluoranthene, pyrene, perylene, benz[a]anthracene, chrysene, benzo[b]fluoranthene, benzo[k]fluoranthene, benzo[a]pyrene, benzo[e]pyrene, indeno[1,2,3‐cd]pyrene, dibenzo[a,h]anthracene, and benzo[ghi]perylene, all of which are target compounds in the USEPA 8270E method [37].

The accelerated solvent extraction (ASE) method was employed for analyte extraction using a Dionex ASE 200 unit. This process included in‐cell cleanup to reduce lipid content, following the protocol proposed by Pinheiro et al. [38], with minor modifications to the extraction cell configuration. Approximately 1 g of wet sample was mixed with 10 g of anhydrous sodium sulfate (Na_2_SO_4_) to facilitate dehydration. The extraction cell was prepared with cellulose filters, 5 g of 5% deactivated silica gel, the Na_2_SO_4_‐treated sample, and 50 ng of *p*‐terphenyl‐d14 (Accustandard) used as a surrogate standard. Remaining cell volume was filled with additional Na_2_SO_4_. The crude extracts were eluted using 90 mL of a 9:1 dichloromethane (DCM) to methanol solution. Extraction conditions included three static cycles of 10 min each at 80°C, with a 90 s purge after each cycle and a rinse volume equal to 75% of the cell's empty capacity to minimize cross‐sample contamination.

Following the initial extraction, crude extracts, blanks, and reference materials were purified using a glass column packed with 10 g of 5% deactivated silica gel, 7 g of 2% deactivated alumina, and approximately 1 g of anhydrous sodium sulfate. Elution was carried out with 80 mL of a 1:1 mixture of DCM and hexane. The eluates were concentrated to a final volume of around 1 mL using a Syncore Analyst concentrator. A solvent exchange to pure hexane was then performed, and 100 ng of deuterated internal standards, including naphthalene‐d8, acenaphthene‐d10, phenanthrene‐d10, chrysene‐d12, and perylene‐d12, were added to each extract.

Quantification of the target contaminants was performed via gas chromatography–mass spectrometry (GC–MS), employing a Trace GC Ultra system coupled with a Thermo Scientific ISQ Single Quadrupole detector. Chromatographic separation was achieved using a DB‐5MS capillary column (30 m × 0.25 mm i.d., 0.25 μm film thickness), operated under a constant helium flow of 1.2 mL min^−1^. The oven temperature program initiated at 50°C for 5 min, increased to 80°C at a rate of 50°C min^−1^, followed by a ramp to 280°C at 6°C min^−1^ with a 4 min hold, and finally rose to 305°C at 12°C min^−1^, holding for an additional 5 min. The mass spectrometer operated in selected ion monitoring (SIM) mode for enhanced sensitivity.

Calibration curves were constructed using 100 ng of internal standard at 10 concentration levels (1, 2, 5, 10, 20, 50, 100, 200, 400, and 1000 ng mL^−1^), covering the PAHs prioritized by the US EPA, along with dibenzothiophene, benzo[e]pyrene, and perylene. All calibration curves exhibited strong linearity, with coefficients of determination (*R* ^2^) greater than 0.99. Analysis of variance (ANOVA) confirmed the statistical significance of the regression models, while the Cochran test verified the homogeneity of variances (homoscedasticity) in the residuals from the least‐squares regression. Limits of Detection (LOD) were calculated as LOD = 3.3(*S*ᵧ/*S*), where *S*ᵧ represents the standard deviation of the response and *S* is the slope of the calibration curve. Limits of Quantification were estimated based on the average sample mass and the lowest validated calibration point. Both are depicted in Table S2 for each determined compound.

Pesticide determinations were conducted in accordance with the USEPA 8081B protocol as adapted by Magalhães et al. [39], which is based on gas chromatography analyses coupled with an electron capture detector (GC–ECD). Calibration of the system was performed using a nine‐point calibration curve with concentrations of 1.0, 5.0, 10, 20, 50, 80, 100, 150, and 200 ng mL^−1^, prepared from a solution containing the following pesticides: alpha‐HCH, beta‐HCH, gamma‐HCH, delta‐HCH, aldrin, isodrin, dieldrin, endrin, heptachlor, heptachlor epoxide A, heptachlor epoxide B, oxychlordane, gamma‐chlordane, alpha‐chlordane, o,p′‐DDE, p,p′‐DDE, o,p′‐DDD, p,p′‐DDD, o,p′‐DDT, p,p′‐DDT, endosulfan I, endosulfan II, methoxychlor, and mirex. Quantification was carried out using the internal standard method, with tetrachlorometaxylene at a fixed concentration of 100 ng mL^−1^ serving as the internal standard.

The instrumental analysis was conducted using gas chromatography with an electron capture detector (GC–ECD). The chromatographic separation was performed on a J&W DB‐5MS capillary column, 30 m in length, with an internal diameter of 0.25 mm and a film thickness of 0.25 μm. The oven temperature program was set as follows: an initial temperature of 60°C held for 3 min, followed by a ramp of 5°C min^−1^ up to 150°C, maintained for 5 min; then ramped at 1°C min^−1^ up to 200°C, and finally increased at 8°C min^−1^ to a final temperature of 300°C, held for 4.5 min. Helium was used as the carrier gas at a constant flow rate of 1.2 mL min^−1^. The injection volume was 1 μL. The method Limit of Detection (LOD) was defined as three times the standard deviation obtained from seven replicates of method blanks. The LOD and LOQ were established as the lowest calibration point divided by the extracted sample mass. A relatively high LOQ was observed due to the low mass of extracted sample. Table S3 summarizes the LOD and LOQ values determined for the investigated pesticides.

### 2.6 Oxidative Stress Biomarker Determinations

Oxidative stress assessments encompassed non‐enzymatic (reduced glutathione and metallothionein) and enzymatic (superoxide dismutase, catalase, and glutathione‐S‐transferase) biomarkers, a radical oxygen species (hydrogen peroxide) and damage end‐points indicative of cytotoxicity (cell membrane lipid peroxidation and protein carbonylation). All analyses were conducted by UV–Vis spectrophotometry.

Reduced glutathione was determined according to Wilhelm Filho et al. [40], through Ellman's reaction with DTNB at 412 nm employing GSH as an external standard. For MT determinations, thermostable proteins were first extracted according to a thermal extraction procedure [41] in a buffer solution containing Tris–HCl 2.0 × 10^−2^ mol L^−1^ pH 8.6, phenylmethylsulfonylfluoride as a protease inhibitor 5.0 × 10^−4^ mol L^−1^, and β‐mercaptoethanol 0.01% as an antioxidant, centrifugation at 20,000 × *g* for 60 min at 4°C, heating at 70°C for 10 min and another centrifugation at 20,000 × *g* for 30 min. The resulting purified supernatants containing the thermostable protein fraction, including MT, were treated with 1 mol L^−1^ HCl containing 4 mmol L^−1^ EDTA, 2 mol L^−1^ NaCl, and 0.43 mmol L^−1^ of 5,5‐dithiobis(2‐nitrobenzoic acid) (DTNB) buffered with 0.2 mol L^−1^ of sodium phosphate, pH 8.0 and sample absorbances were determined at 412 nm [42]. Metallothionein concentrations were estimated using an analytical curve plotted with GSH as an external standard and transformed assuming a stoichiometric relationship of 1 mol MT being the equivalent to 20 mol GSH [43].

Superoxide dismutase (SOD) activities were determined using a commercial kit provided by Cayman Chemical Company (Michigan, USA). The absorbances were determined at 450 nm (*λ*_max_) using an INNO microplate reader (LTEK Co., Moita, Portugal). Glutathione‐S‐transferase was determined according to Habig, Pabst and Jakoby [44], by the development of GS‐DNB at 340 nm for 60 s employing 1‐chloro‐2,4‐dinitrobenzene (CDNB) as substrate, with results expressed as U GST g ptn^−1^. Catalase was determined according to Aebi [45], through the quantification of H_2_O_2_ consumption at 240 nm for 15 s, with results expressed as U CAT g ptn^−1^.

Determination of the oxidative stress endpoint for cytotoxicity, lipid peroxidation, was carried out according to [46]. The samples were first homogenized in 0.1% trichloroacetic acid containing approximately 20% polyvinylpolypyrrolidone, followed by centrifugation at 10,000 × *g* for 1 min. Next, 20% trichloroacetic acid with 0.5% thiobarbituric acid was added, and the samples were incubated for 30 min at 95°C. After cooling on ice and centrifugation at 12,000 × *g* for 10 min, absorbance was measured at 535 nm using a UV–Vis spectrophotometer, with malondialdehyde (MDA) as the external standard.

Sample extraction for protein carbonylation (PTC) determinations was carried out at approximately a 1:16 (tissue: buffer) ratio. The homogenization buffer consisted of 0.1 mol L^−1^ sodium phosphate buffer (pH 6.5) containing 0.25 mol L^−1^ sucrose and 1 mmol L^−1^ EDTA [40]. The homogenates were then centrifuged at 20,000 × *g* for 30 min. Prior to analysis, muscle, brain, spleen, kidney, liver, and gill samples were diluted five‐fold, while gonad, pancreas, and blood samples were diluted 10‐fold. Protein carbonylation was quantified in five‐fold diluted brain and liver samples according to Mesquita et al. [47]. Absorbances were determined at 450 nm using an INNO microplate reader.

Hydrogen peroxide quantification was carried out according to by Alexieva et al. [48]. The samples were homogenized in a 0.1% trichloroacetic acid solution and centrifuged at 10000 *g* for 5 min at 4°C. A 200 μL aliquot of the supernatant was mixed with 200 μL of a 100 mmol L^−1^ potassium phosphate buffer (pH 7.5) and 800 μL of KI 1 mol L^−1^. The tubes were then kept in an ice bath for 1 h in the dark and the absorbances were determined at 390 nm on a UV–Vis spectrophotometer, using a hydrogen peroxide standard curve as an external standard.

## 3 Results and Discussion

The thresher shark examined herein was a juvenile female, measuring 128.8 cm in total length and weighing 4.265 kg.

**FIGURE 2:**
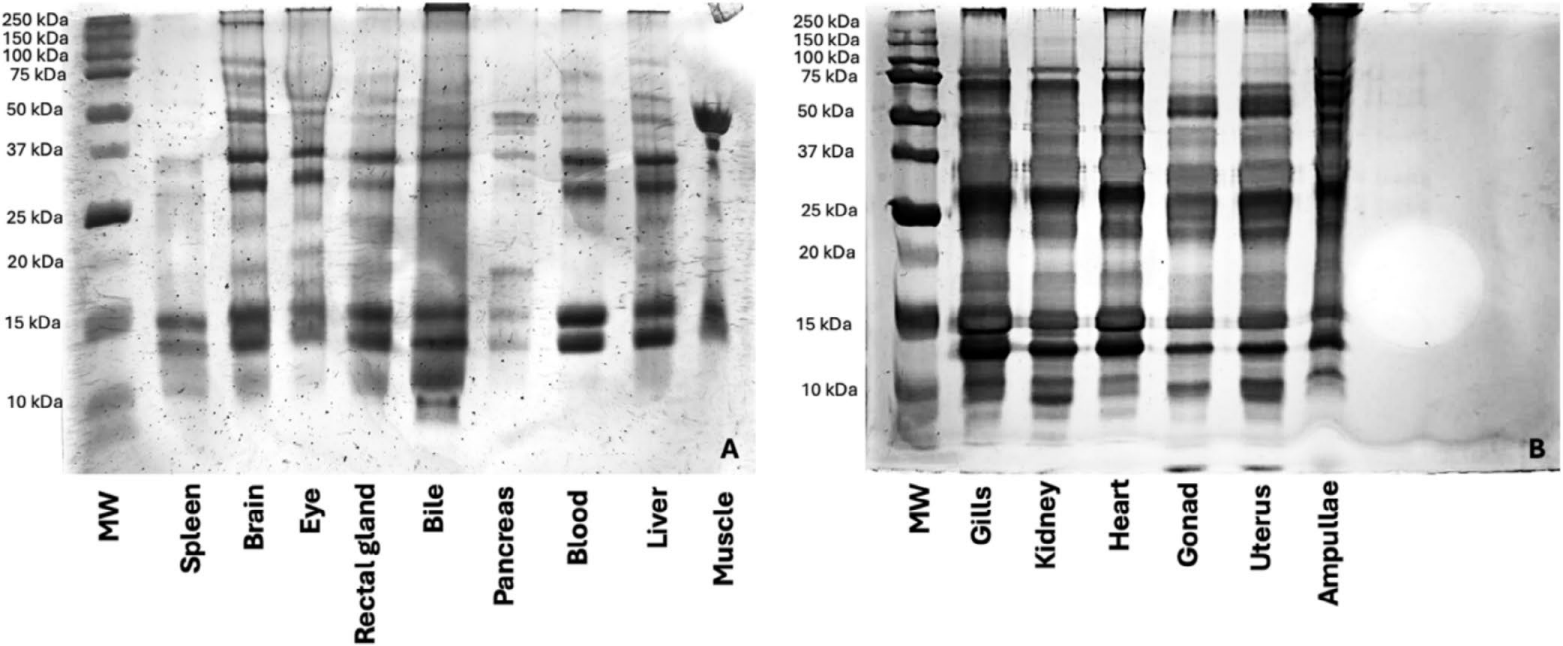
Thermostable electrophoretic protein profiles in the analyzed *Alopias vulpinus* organs and biological fluids.

The thermostable electrophoretic protein profiles in the analyzed thresher shark organs and biological fluids are depicted in Figure 2. Gel images were modified only for clarity, cropping the images and altering saturation, sharpness, brightness and contrast. The original images are available upon request.

Several high molecular weight (HMW) bands are noted in several tissues, such as the gills, kidney, heart, gonad, and uterus, while other organs display mostly medium to low molecular weight (LMW) bands (i.e., spleen, brain, eye, rectal gland, bile, pancrease, blood, liver, and muscle). Bands observed around 15 kDa are consistent with the expected, as the applied heat‐purification step is typically used for MT analysis, which usually ranges at about 14 kDa in different species [49]. While the quantification of MT is specifically based on thiol groups using Ellman's reaction, and MT is likely the dominant protein in the thermostable purified fractions, other thermostable proteins were also detected in all examined common thresher organs. Previous studies on fish liver MTs separated by 1D SDS–PAGE suggest an estimated molecular mass of 7 kDa for the monomer form and 14 kDa for the dimer form [50]. Additionally, research indicate that MT in other fish species may form dimers and tetramers, with molecular masses of 11 kDa in rockfish, 10–12 kDa in dab, and 13–15 kDa in plaice [51, 52]. Therefore, the bands near 15 kDa and slightly below in the common thresher shark may indicate the presence of different structural or functional MT variants (isoforms). Bands noted at around 75 kDa may comprise Matrix metalloproteinase‐2 (MMP‐2), whose inactive zymogen form display around 72 kDa, although species‐specific differences are noted concerning molecular weight [53, 54]. This enzyme is involved in extracellular matrix degradation processes, playing tissue remodeling roles, among other physiological and pathological processes, and has been detected in many different fish organs [53, 54].

This is the first time a 1D SDS–PAGE assessment is conducted for Ampullae of Lorenzini gel samples. The non‐resolution of the protein bands, especially HMW ones, may be due to high salt content, similar to seawater, particularly the presence of ions such as sodium (Na^+^) and potassium (K^+^) [55], resulting in extremely high conductivity [56]. Excess salts can interfere with protein separation by masking protein charges, disrupting SDS binding, or affecting the gel matrix during electrophoresis, leading to poor resolution of protein bands [57]. In addition to Na^+^ and K^+^, other factors such as magnesium (Mg^2+^) or calcium (Ca^2+^), which may also be present in the gel, could further complicate protein migration [58]. To address this issue, method validation should be conducting specifically for this type of sample, employing desalting steps or sample preparation protocols (such as dialysis or protein precipitation) to remove excess salts before running SDS–PAGE gels, ensuring clearer protein band resolution [59].

This study on thermostable proteins using 1D SDS–PAGE represents a preliminary assessment aimed at identifying the presence of these proteins in various common thresher samples. While this approach provides initial insights into protein stability and molecular weight, further assessments are required to gain a comprehensive understanding of the thermostable proteome. Future studies should incorporate additional steps such as 2D gel electrophoresis for better resolution of protein isoforms, mass spectrometry identifications, and bioinformatics assessments for protein modeling and pathway analysis.

The total and thermostable metals in the organs and biological fluids in the analyzed thresher shark organs and biological fluids are depicted in Table 1.

**TABLE 1:** Total and thermostable metals in the organs and biological fluids of a juvenile female thresher shark ( *Alopias vulpinus* ) specimen from Southeastern Brazil.

Sample	Total metals														
	Ti	V	Mn	Fe	Co	Ni	Cu	Zn	As	Se	Rb	Ag	Cd	Hg	Pb
Ampullae of Lorenzini	0.15	0.0175	0.12	5.69	0.003	0.06	0.51	2.19	0.50	< LOQ	0.12	< LOQ	< LOQ	< LOQ	0.071
Eyes	0.76	0.0173	0.48	18.41	0.006	0.05	0.48	16.66	2.55	< LOQ	0.26	< LOQ	< LOQ	< LOQ	< LOQ
Bile	0.24	0.0098	0.30	10.76	< LOQ	< LOQ	6.71	5.13	0.62	0.36	0.45	0.025	< LOQ	< LOQ	< LOQ
Fin	3.58	0.1479	1.93	90.13	0.097	0.46	1.78	26.20	3.48	1.17	0.23	0.002	0.010	0.02	0.242
Muscle	0.73	0.0274	0.07	1.70	< LOQ	< LOQ	0.47	12.85	0.40	< LOQ	0.80	< LOQ	< LOQ	0.14	< LOQ
Brain	0.84	0.0148	0.08	5.75	0.005	< LOQ	1.01	5.79	0.45	0.22	0.36	< LOQ	< LOQ	0.01	< LOQ
Rectal gland	0.54	0.0152	0.32	39.29	0.005	< LOQ	1.06	11.40	0.87	0.63	0.73	0.001	< LOQ	0.02	< LOQ
Pancreas	0.80	0.0201	1.15	41.66	0.004	< LOQ	1.81	44.58	0.68	1.17	0.73	0.002	0.004	0.01	< LOQ
Gills	1.29	0.1034	1.29	180.68	0.092	0.08	1.49	18.29	1.38	0.96	0.53	0.003	0.005	0.22	0.067
Heart	0.58	0.0181	0.15	77.18	0.007	< LOQ	2.07	15.49	0.61	0.45	0.46	< LOQ	< LOQ	0.08	< LOQ
Spleen	0.94	0.019	0.42	66.96	0.004	< LOQ	0.93	16.10	0.90	0.85	0.66	< LOQ	0.002	0.01	< LOQ
Uterus	0.60	0.0179	0.39	20.99	0.005	< LOQ	0.80	7.97	0.52	0.66	0.51	< LOQ	0.003	< LOQ	< LOQ
Gonads	0.65	0.015	0.61	79.56	0.004	0.15	0.83	16.79	0.84	0.64	0.56	< LOQ	0.001	0.02	< LOQ
Kidneys	0.69	0.015	0.86	41.49	0.005	< LOQ	1.04	12.46	0.65	0.62	0.54	< LOQ	0.001	0.02	< LOQ
Liver	0.60	0.0125	1.02	157.80	0.0083	< LOQ	3.28	19.02	21.63	< LOQ	0.40	0.007	0.002	0.02	< LOQ
Blood	0.35	0.0133	0.11	59.65	0.0052	< LOQ	0.51	6.07	0.51	0.32	0.47	< LOQ	< LOQ	0.04	< LOQ

Sample	La	Ce	Pr	Nd	Sm	Eu	Gd	Tb	Dy	Ho	Er	Tm	Yb	Lu
Ampullae of Lorenzini	0.0031	0.0073	0.0003	< LOQ	0.0009	0.0002	< LOQ	< LOQ	0.0006	< LOQ	0.0002	< LOQ	0.00004	< LOQ
Eyes	< LOQ	0.0026	< LOQ	< LOQ	< LOQ	< LOQ	< LOQ	< LOQ	< LOQ	< LOQ	< LOQ	< LOQ	0.00004	< LOQ
Bile	< LOQ	< LOQ	< LOQ	< LOQ	0.0003	< LOQ	< LOQ	< LOQ	< LOQ	< LOQ	< LOQ	< LOQ	0.00004	< LOQ
Fin	0.0123	0.0286	0.0031	< LOQ	0.0041	0.0003	0.0034	0.0004	0.0015	0.0003	0.0005	0.0001	0.00063	< LOQ
Muscle	< LOQ	< LOQ	< LOQ	< LOQ	0.0002	< LOQ	< LOQ	< LOQ	< LOQ	< LOQ	< LOQ	< LOQ	0.00004	< LOQ
Brain	< LOQ	< LOQ	< LOQ	< LOQ	< LOQ	< LOQ	< LOQ	< LOQ	< LOQ	< LOQ	< LOQ	< LOQ	0.00005	< LOQ
Rectal gland	< LOQ	< LOQ	< LOQ	< LOQ	0.0004	< LOQ	< LOQ	< LOQ	< LOQ	< LOQ	0.0002	0.0001	0.00004	0.0002
Pancreas	< LOQ	< LOQ	< LOQ	< LOQ	0.0002	< LOQ	< LOQ	< LOQ	< LOQ	< LOQ	< LOQ	< LOQ	0.00004	< LOQ
Gills	0.0299	0.0778	0.0068	< LOQ	0.0052	0.0011	0.0106	0.0006	0.0085	<LOQ	0.0018	0.0005	0.00342	0.0004
Heart	< LOQ	0.0008	< LOQ	< LOQ	0.0004	0.0002	< LOQ	< LOQ	< LOQ	< LOQ	< LOQ	< LOQ	0.00005	< LOQ
Spleen	< LOQ	< LOQ	< LOQ	< LOQ	0.0005	< LOQ	< LOQ	< LOQ	< LOQ	< LOQ	< LOQ	< LOQ	0.00019	< LOQ
Uterus	< LOQ	< LOQ	< LOQ	< LOQ	0.0003	< LOQ	< LOQ	< LOQ	< LOQ	< LOQ	< LOQ	< LOQ	0.00005	< LOQ
Gonads	< LOQ	< LOQ	< LOQ	< LOQ	0.0002	< LOQ	< LOQ	< LOQ	< LOQ	< LOQ	< LOQ	< LOQ	0.00004	< LOQ
Kidneys	< LOQ	< LOQ	< LOQ	< LOQ	0.0007	0.0002	< LOQ	< LOQ	< LOQ	< LOQ	< LOQ	< LOQ	0.00004	< LOQ
Liver	0.0011	0.0031	< LOQ	< LOQ	< LOQ	0.0002	< LOQ	< LOQ	< LOQ	< LOQ	< LOQ	< LOQ	0.00017	< LOQ
Blood	< LOQ	< LOQ	< LOQ	< LOQ	0.0002	< LOQ	< LOQ	< LOQ	< LOQ	< LOQ	< LOQ	< LOQ	0.00005	< LOQ

Sample	Thermostable metals														
	Ti	V	Mn	Fe	Co	Ni	Cu	Zn	As	Se	Rb	Ag	Cd	Hg	Pb
Ampullae of Lorenzini	0.05	< LOQ	< LOQ	2.54	< LOQ	< LOQ	< LOQ	0.07	0.02	< LOQ	0.02	< LOQ	< LOQ	< LOQ	0.006
Eyes	0.11	< LOQ	< LOQ	3.06	< LOQ	< LOQ	< LOQ	0.10	0.08	< LOQ	0.07	< LOQ	< LOQ	< LOQ	0.005
Bile	0.25	< LOQ	0.1041	4.97	< LOQ	< LOQ	2.02	0.81	0.26	< LOQ	0.21	< LOQ	< LOQ	< LOQ	0.006
Fin	0.13	< LOQ	< LOQ	3.48	< LOQ	< LOQ	< LOQ	< LOQ	0.15	< LOQ	0.06	< LOQ	< LOQ	< LOQ	0.006
Muscle	0.35	< LOQ	< LOQ	2.00	< LOQ	< LOQ	< LOQ	1.63	0.05	< LOQ	0.24	< LOQ	< LOQ	< LOQ	0.004
Brain	0.16	< LOQ	< LOQ	2.40	< LOQ	< LOQ	0.17	0.26	0.08	< LOQ	0.10	< LOQ	< LOQ	< LOQ	< LOQ
Rectal gland	0.20	< LOQ	< LOQ	3.18	< LOQ	< LOQ	< LOQ	0.27	0.10	< LOQ	0.14	< LOQ	< LOQ	< LOQ	< LOQ
Pancreas	0.31	< LOQ	0.2965	2.35	< LOQ	< LOQ	0.43	7.65	0.17	< LOQ	0.32	< LOQ	< LOQ	< LOQ	< LOQ
Gills	0.17	< LOQ	< LOQ	6.62	< LOQ	< LOQ	< LOQ	0.19	0.08	< LOQ	0.14	0.005	< LOQ	< LOQ	0.005
Heart	0.19	< LOQ	< LOQ	6.50	< LOQ	< LOQ	< LOQ	0.17	0.09	< LOQ	0.14	< LOQ	< LOQ	< LOQ	< LOQ
Spleen	0.32	< LOQ	< LOQ	3.80	< LOQ	< LOQ	0.09	0.33	0.11	< LOQ	0.17	< LOQ	< LOQ	< LOQ	< LOQ
Uterus	0.27	< LOQ	0.0555	4.65	< LOQ	< LOQ	0.09	0.43	0.10	0.11	0.17	< LOQ	< LOQ	< LOQ	< LOQ
Gonads	0.24	< LOQ	< LOQ	4.03	< LOQ	< LOQ	0.09	0.76	0.10	< LOQ	0.17	< LOQ	< LOQ	< LOQ	< LOQ
Kidneys	0.17	< LOQ	0.1607	2.74	< LOQ	< LOQ	< LOQ	0.14	0.07	< LOQ	0.14	< LOQ	< LOQ	< LOQ	< LOQ
Liver	0.16	< LOQ	0.1378	14.16	< LOQ	< LOQ	0.55	1.23	0.11	< LOQ	0.12	< LOQ	< LOQ	< LOQ	< LOQ
Blood	0.20	< LOQ	< LOQ	7.59	< LOQ	< LOQ	< LOQ	0.25	0.11	< LOQ	0.16	< LOQ	< LOQ	< LOQ	< LOQ

Sample	La	Ce	Pr	Nd	Sm	Eu	Gd	Tb	Dy	Ho	Er	Tm	Yb	Lu
Ampullae of Lorenzini	< LOQ	0.0023	0.0028	< LOQ	0.0030	< LOQ	< LOQ	< LOQ	< LOQ	< LOQ	< LOQ	< LOQ	< LOQ	< LOQ
Eyes	< LOQ	0.0022	0.0028	< LOQ	0.0031	< LOQ	< LOQ	< LOQ	< LOQ	< LOQ	< LOQ	< LOQ	< LOQ	< LOQ
Bile	< LOQ	0.0022	0.0028	< LOQ	0.0033	< LOQ	< LOQ	< LOQ	< LOQ	< LOQ	< LOQ	< LOQ	< LOQ	< LOQ
Fin	< LOQ	0.0024	0.0034	< LOQ	0.0036	< LOQ	< LOQ	< LOQ	< LOQ	< LOQ	< LOQ	< LOQ	< LOQ	< LOQ
Muscle	< LOQ	0.0021	0.0029	< LOQ	0.0032	< LOQ	< LOQ	< LOQ	< LOQ	< LOQ	< LOQ	< LOQ	< LOQ	< LOQ
Brain	< LOQ	0.0018	0.0027	< LOQ	0.0028	< LOQ	< LOQ	< LOQ	< LOQ	< LOQ	< LOQ	< LOQ	< LOQ	< LOQ
Rectal gland	< LOQ	0.0020	0.0029	< LOQ	0.0031	< LOQ	< LOQ	< LOQ	< LOQ	< LOQ	< LOQ	< LOQ	< LOQ	< LOQ
Pancreas	< LOQ	0.0019	0.0027	< LOQ	0.0030	< LOQ	< LOQ	< LOQ	< LOQ	< LOQ	< LOQ	< LOQ	< LOQ	< LOQ
Gills	< LOQ	0.0022	0.0028	< LOQ	0.0030	< LOQ	< LOQ	< LOQ	< LOQ	< LOQ	< LOQ	< LOQ	< LOQ	< LOQ
Heart	< LOQ	0.0016	0.0024	< LOQ	0.0026	< LOQ	< LOQ	< LOQ	< LOQ	< LOQ	< LOQ	< LOQ	< LOQ	< LOQ
Spleen	< LOQ	0.0020	0.0027	< LOQ	0.0029	< LOQ	< LOQ	< LOQ	< LOQ	< LOQ	< LOQ	< LOQ	< LOQ	< LOQ
Uterus	< LOQ	0.0021	0.0028	< LOQ	0.0031	< LOQ	< LOQ	< LOQ	< LOQ	< LOQ	< LOQ	< LOQ	< LOQ	< LOQ
Gonads	< LOQ	0.0018	0.0026	< LOQ	0.0028	< LOQ	< LOQ	< LOQ	< LOQ	< LOQ	< LOQ	< LOQ	< LOQ	< LOQ
Kidneys	< LOQ	0.0017	0.0025	< LOQ	0.0027	< LOQ	< LOQ	< LOQ	< LOQ	< LOQ	< LOQ	< LOQ	< LOQ	< LOQ
Liver	< LOQ	0.0020	0.0028	< LOQ	0.0033	< LOQ	< LOQ	< LOQ	< LOQ	< LOQ	< LOQ	< LOQ	< LOQ	< LOQ
Blood	< LOQ	0.0021	0.0028	< LOQ	0.0030	< LOQ	< LOQ	< LOQ	< LOQ	< LOQ	< LOQ	< LOQ	< LOQ	< LOQ

*Note:* < LOQ indicate values below the limit of quantification. Data are expressed as mg kg^−1^ wet weight (w.w.).

Most metals were present in significantly higher concentrations in the total fraction compared to the thermostable fraction across several organs. Since the thermostable fraction represents proteins that remain after heat treatment, the lower metal concentrations here suggest that, while some metals are associated with MTs in the thermostable fraction, a significant pool of metals may remain unbound or associated with other cellular components, such as non‐thermostable proteins. On the other hand, several elements were present below the quantifiable limit in the thermostable fractions across the sampled organs, indicating probable effective detoxification and removal from this fraction, potentially through MTs actively engaged in detoxifying metals that are not yet sequestered in a thermostable form, reflecting the dynamic nature of metal handling in the analyzed tissues. Future studies should, therefore, focus on isolating and characterizing the MTs from these organs to better understand their specific metal‐binding affinities and capacities, as well as their overall contribution to metal detoxification and regulation in *A. vulpinus*.

Mercury is the most frequently analyzed contaminant in *Alopias* spp., with consistently high total mercury (THg) and methylmercury (MeHg) levels reported across the genus, particularly in muscle [60, 61, 62]. Studies from the eastern tropical Pacific, Galápagos, and Colombian coasts collectively show that *A. pelagicus* and *A. superciliosus* accumulate substantially more Hg in muscle than in liver or blood, reflecting the high trophic position and long‐range movements of these pelagic predators [60, 61]. Although absolute concentrations vary among regions, the general pattern of pronounced muscle enrichment and comparatively lower internal tissue concentrations is robust across the literature.

Other elements exhibit species‐ and tissue‐specific patterns. For example, Zn and Cu tend to dominate the elemental profile of *Alopias* muscle [63, 64], reflecting their essential metabolic roles, while Cd, Pb, and Hg are typically present at lower concentrations. In contrast, some Mediterranean and Mexican studies have reported high As, Se, or radiocesium, highlighting the influence of local environmental conditions [65, 66]. Selenium often co‐occurs with Hg, and several studies emphasize the toxicological relevance of the Se:Hg molar ratio, with values > 1 commonly interpreted as indicative of a potential protective effect [63, 65], although exceptions are also noted, in which adult thresher sharks exhibited ratios < 1, suggesting insufficient Se‐mediated detoxification [66].

Comparative studies across shark species indicate that *A. vulpinus* generally accumulates less Hg than more apex‐oriented taxa such as *Carcharodon carcharias* or *Isurus oxyrinchus* , reinforcing interspecific differences in trophic ecology and exposure pathways [67]. Multi‐tissue assessments further show that liver, gills, and gonads can accumulate specific metals according to their physiological functions [68], highlighting the importance of multi‐compartment analyses when evaluating trace element burdens. Maternal–embryonic analyses also reveal differential accumulation patterns and highlight the potential role of Se in early‐life protection [69].

Together, these comparative patterns show that the contaminant levels measured in our specimen fall well below the ranges previously reported for *Alopias* spp., indicating comparatively lower levels in southeastern Brazil compared to the eastern Pacific, Mediterranean, or Mexican Pacific, and likely reflecting the specimen's juvenile age, limited trophic accumulation, and local environmental conditions.

Concerning organic contaminants, only liver and muscle could be analyzed herein due to lack of organ mass. In this sense, although a non‐targeted screening for several pesticides was carried out (a‐BHC, g‐BHC, b‐BHC, d‐BHC, Heptachlor, Aldrin, a‐Chlordane, Isodrin, Endosulfan, o,p′‐DDE + G‐CHLORDAN, Heptachloroepoxide A, Oxychlordane, heptachloroepoxide B, Dieldrin, p,p′‐DDE, o,p′‐DDD, Endrin, Endosulfan II, p,p′‐DDD, o,p′‐DDT, Methoxychlor, p,p′‐DDT and), only p,p′‐DDT and Mirex were detected; however, at very high concentrations of 59.81 ng g^−1^ (w.w.) in liver and 95.42 ng g^−1^ (w.w.) in muscle, respectively.

Regarding PAH, however, the situation was quite different, with many PAH detected in both tissues, as depicted in Table 2.

**TABLE 2:** Polycyclic aromatic hydrocarbons in the organs and biological fluids of a juvenile female *Alopias vulpinus* specimen from southeastern Brazil.

Compound	Liver	Muscle
∑16PAH	39.16	72.67
∑40PAH	49.44	142.35
∑16PAH–N	19.86	34.93
∑40PAH–N	30.14	104.61
Naphthalene	19.29	37.74
Acenaphthylene	0.19	2.83
Acenaphthene	0.34	2.63
Fluorene	0.90	4.49
Dibenzothiophene	1.11	< LOQ
Phenanthrene	5.21	7.62
Anthracene	1.35	6.07
Fluoranthene	1.81	6.17
Pyrene	2.84	3.54
C1‐fluoranthene–pyrene	2.49	< LOQ
C2‐fluoranthene–pyrene	3.56	< LOQ
C3‐fluoranthene–pyrene	0.10	< LOQ
Benzo(c)fluoranthene	< LOQ	< LOQ
Benzo(a)anthracene	1.28	< LOQ
Chrysene	1.20	1.58
C1‐chrysene	1.53	< LOQ
C2‐chrysene	< LOQ	< LOQ
Benzo(b)fluoranthene	< LOQ	< LOQ
Benzo(k) fluoranthene	< LOQ	< LOQ
Benzo(e)pyrene	1.49	< LOQ
Benzo(a)pyrene	1.65	< LOQ
Perylene	< LOQ	< LOQ
Indene(1,2,3‐c,d)pyrene	< LOQ	< LOQ
Dibenzo(a,h)anthracene	3.09	< LOQ
Benzo(g,h,i)perylene	< LOQ	< LOQ
LMW compounds	27.28	61.38
HMW compounds	11.88	11.29
LMH/HMH	2.30	5.44

*Note:* Data are expressed as ng g^−1^ wet weight (w.w.).

Abbreviations: HMW, high molecular weight polycyclic aromatic hydrocarbons; LMH/HMH, ratio of low molecular to high molecular weight polycyclic aromatic hydrocarbons; LMW, low molecular weight polycyclic aromatic hydrocarbons.

Contrary to the expected, most PAHs were present in higher concentrations in muscle instead of liver. This is of concern, indicating potential bioaccumulation processes in place in muscle tissue, due to an overwhelmed hepatic detoxification system [70], the main xenobiotic metabolizing organ in vertebrates [71, 72]. This may be due to chronic exposure to sources of these compounds. Benzo(g,h,i)perylene, C2‐Chrysene, Benzo(b)fluoranthene, Benzo(k)fluoranthene, and Benzo(c)fluoranthene were not detected in either organ, while Dibenzothiophene, C1‐Fluoranthene–pyrene, C2‐Fluoranthene‐pyrene, C3‐Fluoranthene‐pyrene, Benzo(a)anthracene, Benzo(e)pyrene, Benzo(a)pyrene, and Dibenzo(a,h)anthracene were only detected in liver.

Studies on organic pollutants in *Alopias* spp. are limited and have focused primarily on PCBs, PBDEs, and pesticides (DDTs), with no prior assessments concerning PAHs in the genus [73, 74, 75, 76]. Reported concentrations of legacy POPs vary widely across species and regions, but most studies consistently show low‐to‐moderate levels of DDTs and PCBs in *Alopias* muscle, with values generally below 2 mg k g^−1^ w.w. for both groups [67, 76]. Even higher reported loads of DDXs (DDT metabolites) and other pesticides in *A. vulpinus* liver [75] remain orders of magnitude above those detected herein.

In contrast, the non‐target screening carried out herein detected only two pesticides, p,p'‐DDT and Mirex, yet at unexpectedly high concentrations in liver and muscle, respectively. Although these values remain substantially lower than the mg‐level burdens reported for *Alopias* spp. from the northeastern Pacific, they are high relative to the generally low ng‐to‐sub‐ng levels observed in *A. superciliosus* sampled off the coast of Brazil [76] and comparable to the PBDE/PCB levels reported in Korean specimens [77]. These findings suggest localized exposure to DDT‐related residues and Mirex, both historically used and environmentally persistent in parts of Brazil.

The present study also provides the first PAH dataset for *Alopias* spp., revealing high accumulation. Detected ΣPAH concentrations were substantially higher in muscle (72.67 ng g^−1^; Σ40PAH = 142.35 ng g^−1^) than in liver, a pattern contrary to expectations for metabolically processed contaminants. Such muscle‐dominant enrichment suggests chronic exposure and incomplete hepatic detoxification, consistent with evidence that PAHs can overwhelm the xenobiotic metabolism in fish [70, 72]. Several high molecular weight PAHs, including benzo(a)pyrene, benzo(e)pyrene, and dibenzo(a,h)anthracene, were detected exclusively in liver, while others were absent in both tissues, indicating selective exposure and metabolic handling.

Overall, the POP concentrations reported herein fall below the high burdens reported for *Alopias* spp. in the Pacific, but exceed or differ from values reported for the western South Atlantic, particularly regarding PAH profiles and the unexpected tissue distribution of several compounds, pointing to region‐specific contamination sources affecting this juvenile shark and suggesting early evidence of bioaccumulation processes even at an early life stage.

**FIGURE 3:**
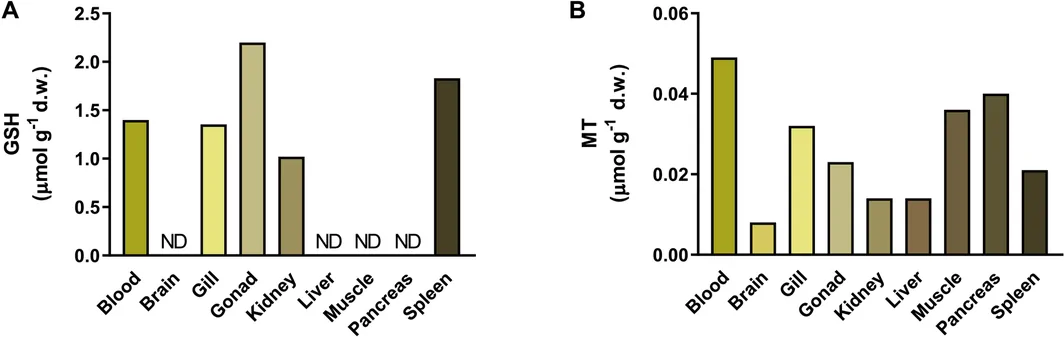
Non‐enzymatic (A) reduced glutathione (GSH) and (B) metallothionein (MT) profiles in different *Alopias vulpinus* tissues. Data without error bars refer to analyses with *n* = 1.

**FIGURE 4:**
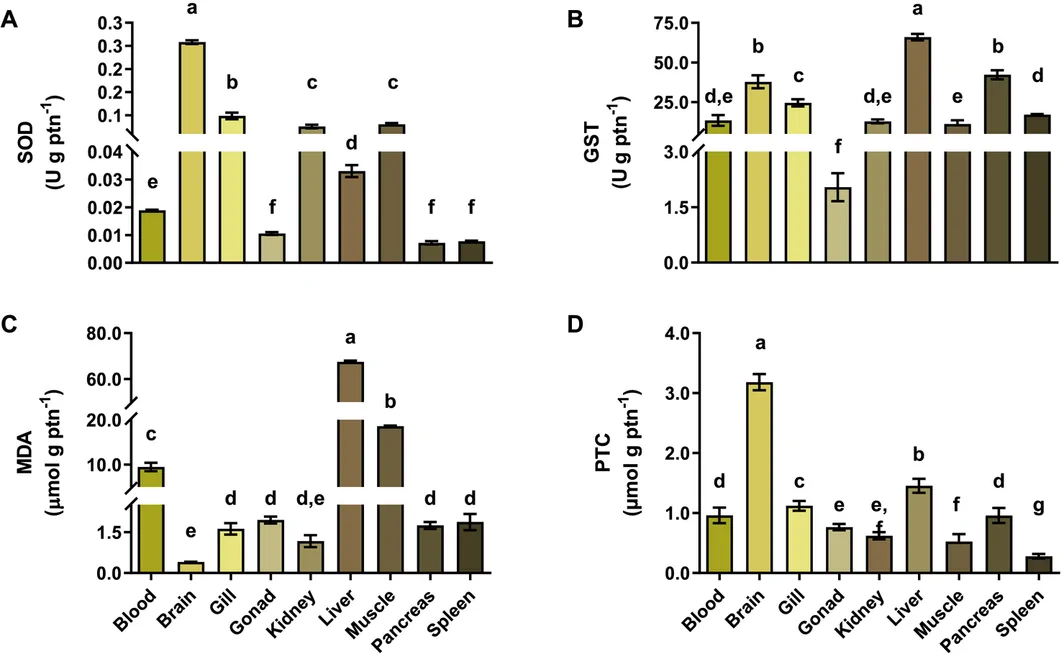
Enzymatic biomarkers (A) superoxide dismutase (SOD) and (B) glutathione‐S‐transferase (GST) profiles, and cytotoxicity biomarkers (C) malondialdehyde (MDA) and (D) protein carbonylation (PTC) in different *Alopias vulpinus* tissues. The bars represent the means ± standard deviations. The same letters above the bars represent the absence of statistical significance, while different letters represent statistical differences between the analyzed tissues.

Biomarker results are depicted in Figures 3 and 4. Few studies have evaluated oxidative stress or antioxidant biomarkers in *Alopias* spp., with existing research largely limited to contaminant burdens, bioaccumulation patterns, and maternal transfer rather than physiological responses to pollution. Thus, the present results offer some of the first insight into tissue‐specific antioxidant dynamics in the genus.

Overall, biomarker patterns varied strongly among tissues, reflecting both intrinsic metabolic differences and potential sublethal responses to contaminant exposure. Catalase activity was undetectable in all tissues. Whether this reflects genuinely low catalase activity in juvenile *A. vulpinus* or methodological constraints cannot be determined without positive controls or alternative assays, and future studies are required to clarify this result.

Superoxide dismutase (SOD), glutathione‐S‐transferase (GST), malondialdehyde (MDA), and carbonylated proteins (PTC) are all reported alongside their standard deviations and were part of statistical analyses, unlike H_2_O_2_, reduced glutathione (GSH), and metallothionein (MT), in which sample masses were not enough for some of the analyses.

Reduced glutathione (GSH), the main non‐enzymatic antioxidant in vertebrates, was high in the gonads (2.19 μmol g ptn^−1^), spleen (1.83 μmol g^−1^), blood (1.39 μmol g^−1^), gills (1.35 μmol g^−1^), and kidneys (1.02 μmol g^−1^) (Figure 3A), and absent from metabolically important organs such as liver, muscle, brain, and pancreas. This pattern may indicate localized depletion due to oxidative demands in these tissues, consistent with high ROS production observed in other shark species exposed to contaminants.

Metallothionein was present in all tissues, with highest levels in blood (0.05 μmol g ptn^−1^), pancreas (0.04 μmol g^−1^), and muscle (0.03 μmol g^−1^). These metalloproteins are traditionally recognized for their role in metal detoxification. Supporting this, Hauser‐Davis et al. [78] reported that As and Zn levels in the liver and muscle of blue sharks ( *Prionace glauca* ) were associated with MT induction, particularly in hepatic tissue. However, growing evidence supports their involvement in mitigating oxidative stress induced by organic pollutants such as PAHs. These compounds undergo enzymatic biotransformation via the cytochrome P450 system, producing ROS and electrophilic intermediates [79]. Such reactive products disrupt cellular redox balance and stimulate MT upregulation as part of the antioxidant defense system [80]. In fact, studies in fish have shown that MT gene and protein expression levels rise in response to PAH exposure, even in the absence of high metal concentrations, suggesting a broader cytoprotective role of MTs against organic contaminants [81]. Although based on a single juvenile individual, the high MT concentrations detected in the blood, pancreas, and muscle of *A. vulpinus* may tentatively indicate activation of metal‐binding pathways in tissues involved in metabolic regulation and detoxification, a pattern consistent with previous observations in other elasmobranchs.

Figure 4 depicts the activity of the enzymatic biomarkers SOD and GST and cytotoxicity biomarkers lipid peroxidation and protein carbonylation results.

Superoxide dismutase (SOD), the enzyme responsible for the dismutation of the superoxide radical (O_2_˙^−^) into H_2_O_2_, was highest in the brain (0.2581 U g ptn^−1^), followed by gills (0.0985 U g ptn^−1^), pancreas (0.0805 U g ptn^−1^), kidneys (0.0757 U g ptn^−1^), and muscle (0.033 U g ptn^−1^) (Figure 4A). These tissues typically experience high oxygen fluxes or metabolic activity and often exhibit strong basal antioxidant defenses. Comparisons with available shark studies suggest that SOD levels in *A. vulpinus* fall within or above the ranges reported for other species, including *C. carcharias* and *Rhizoprionodon terraenovae* , where SOD modulation has been linked to metal exposure, particularly As, Pb and Zn [82, 83]. Given the measurable levels of these contaminants in the present specimen, similar exposure‐related upregulation cannot be ruled out, although further assessments are required. Furthermore, several factors may influence enzymatic activity, including chemical contamination, age, temperature, and salinity, all of which can modulate oxidative stress responses and antioxidant defenses in fish, potentially affecting their physiological resilience and sensitivity to environmental stressors [84, 85, 86, 87].

Exposure to As, for example, is known to increase the production of several ROS such as O_2_˙^−^, OH˙ and H_2_O_2_ [88]. This metalloid can also affect mitochondrial function by binding to thiol (–SH) groups of antioxidant enzymes, such as succinate dehydrogenase, directly affecting the electron transport chain during cellular respiration. This enhances electron leakage and superoxide (O_2_˙^−^) formation [89]. Additionally, As can activate NADPH oxidase, leading to excessive production of O_2_˙^−^. Another important pathway through which As interferes with the antioxidant system occurs during its biotransformation, where As^+3^ is converted into monomethylarsonic acid and dimethylarsinic acid by the enzyme arsenic methyltransferase [90]. These reactions may generate ROS, including O_2_˙^−^ and H_2_O_2_, through redox reactions involving thiol groups and redox‐active Fe [90, 91]. The exact mechanism of ROS formation associated to As, however, remains an active area of research. An intriguing pathway for H_2_O_2_ production was proposed by Valko et al. [91], involving the spontaneous oxidation of As^+3^ and As^+5^ in the presence of H_2_O and O_2_. Although H_2_O_2_ is not a free radical, in organisms with Fe overload, high cellular concentrations of Fe^2+^ can lead to a deleterious effect by catalyzing the decomposition of H_2_O_2_ through the Fenton and Haber‐Weiss reactions, generating highly reactive hydroxyl radicals [91].

GST activity was highest in the liver (65.9 U g ptn^−1^) and pancreas (42.3 U g ptn^−1^), aligning with their primary roles in xenobiotic biotransformation [71, 72]. The presence of GST in all analyzed tissues indicates an active phase II detoxification capacity even in a juvenile individual, consistent with the need to metabolize both endogenous and contaminant‐derived electrophiles.

Hydrogen peroxide levels, one of the main ROS, were the highest in the blood (1.93 μmol g ptn^−1^), gills (2.04 μmol g ptn^−1^), and liver (1.33 μmol g ptn^−1^), suggesting greater ROS accumulation in tissues involved in oxygen transport or xenobiotic metabolism. In contrast, low H_2_O_2_ levels in the brain despite high SOD activity suggest efficient downstream detoxification, whereas, the absence of detectable H_2_O_2_ in the gonads and spleen may reflect lower oxidative demand or sample‐mass limitations. It is important to note that this compound was measured to provide a descriptive insight into oxidative processes in *Alopias* spp., offering preliminary baseline information that may be useful for comparison in future studies even though it is not considered a core biomarker, unlike SOD, reported further ahead.

Lipid peroxidation (MDA) was highest in the liver, indicating membrane damage consistent with depleted GSH and heightened ROS production. This pattern parallels observations in *Isurus oxyrinchus* [88], reinforcing that hepatic tissue can be vulnerable to oxidative challenges even in pelagic sharks. In contrast, low MDA in the brain, spleen, and blood suggests effective antioxidant protection in these tissues.

Finally, PTC was most pronounced in the brain and liver, supporting the inference that oxidative injury is concentrated in metabolically active organs. Lower PTC levels in muscle, kidney, and spleen indicate comparatively reduced oxidative damage.

## 4 Conclusion

This case study offers a comprehensive view of chemical contamination and oxidative stress biomarkers in an *A. vulpinus* specimen off the southeastern coast of Brazil. Persistent pesticides (p,p′‐DDT and Mirex), low molecular weight PAHs, and metals were detected, with differing patterns between total and thermostable fractions, suggesting active compartmentalization and detoxification of several of these contaminants. Protein electrophoresis revealed bands likely corresponding to metallothionein (~15 kDa) and enzymes, such as MMP‐2 (~75 kDa). At the same time, biomarkers of oxidative stress showed signs of ROS buildup and antioxidant responses, varying by tissue type. The findings also suggest a possible species‐specific sensitivity of *A. vulpinus* to chemical pollutants compared to different species.

It is, however, important to note that the assessment was based on a single individual. While data from this individual provide valuable preliminary insights, such a low sample number significantly restricts the ability to generalize findings to the species level. Inter‐individual variability, including differences in age, sex, health status, and environmental exposure, cannot be accounted for, making it difficult to draw robust conclusions or identify consistent patterns across the population. Therefore, any interpretations must be considered preliminary, and broader conclusions regarding species‐wide trends or physiological responses are precluded until data from a larger, more representative sample are available.

Still, the integrated approach used in this study provides a valuable tool for generating baseline data for *Alopias* spp., combining biochemical, molecular, and tissue‐specific assessments to assess early physiological stress and sublethal effects of contaminant exposure that may influence key fitness parameters, including growth, energy allocation, reproduction, and immune competence, as chemical pollution can lead to impaired gonadal development, reduced fecundity, and increased susceptibility to disease. Integrating these physiological indicators with ecological and life‐history data allows for more informed conservation strategies, such as prioritizing critical habitats, mitigating pollutant sources, and establishing monitoring programs to track population health over time, providing evidence to guide management policies aimed at preserving population viability and supporting the long‐term survival of this vulnerable shark species.

## Author Contributions

**Sidney Fernandes Sales Junior:** methodology, investigation, data curation, formal analysis, writing – original draft, and writing – review and editing. **Amanda Pontes Lopes:** methodology, data curation, formal analysis, and writing – review and editing. **Heloíse Martins de Souza and Regina Fonseca:** methodology, data curation, and formal analysis. **Enrico Mendes Saggioro:** methodology, investigation, data curation, validation, supervision, resources, writing – original draft, and writing – review and editing. **Francielli Monteiro Casanova and Kamila Cezar Gramlich:** methodology, investigation, data curation, and formal analysis. **Tatiana Dillenburg Saint'Pierre, Carlos German Massone, and Renato Carreira:** methodology, investigation, data curation, supervision, resources, validation, writing – original draft, and writing – review and editing. **Rachel Ann Hauser‐Davis:** conceptualization, methodology, investigation, data curation, formal analysis, supervision, project administration, resources, visualization, validation, writing – original draft, and writing – review and editing.

## Funding

This work was supported by the Carlos Chagas Filho Foundation for Research Support of the State of Rio de Janeiro (FAPERJ) (Apoio ao Jovem Pesquisador Fluminense com Vínculo em ICTS do Estado do RJ 2021, Grant E‐26/210.300/20222; Jovem Cientista do Nosso Estado 2021–2024, Grant E‐26/201.270/202; Programa Pensa Rio—Apoio ao estudo de temas relevantes e estratégicos para o estado do Rio de Janeiro 2025, Grant E‐26/210.723/2025), as well as the Brazilian National Council of Scientific and Technological Development (productivity grants, Grants 308811/2021‐6 and 301422/2025‐7).

## Supporting information


**Table S1:** tox70026‐sup‐0001‐TableS1‐S3.docx.
**Table S2:** tox70026‐sup‐0001‐TableS1‐S3.docx.
**Table S3:** tox70026‐sup‐0001‐TableS1‐S3.docx.

## Data Availability

The authors confirm that the data supporting the findings of this study are available within the article and its Supporting Information. Raw data that support the findings of this study are available from the corresponding author upon reasonable request.
