# Supplementary material for: Organic and Inorganic Pollutants, Oxidative Stress Biomarkers, and Electrophoretic Protein Profiles in an Endangered Thresher Shark (Alopias vulpinus) From Southeastern Brazil: A Case Study
Source: Environ Toxicol. 2026 Jan 15;41(10):833–48. doi: 10.1002/tox.70026 (PMC13537363; doi:10.1002/tox.70026)
Supplement: Supplementary file 1 [file TOX-41-833-s001.docx]

Supplementary material

Table S1. Limits of Detection (LOD) and Limits of Quantification (LOQ) for each analyzed metal and metalloid determined in the thresher shark (*Alopias vulpinus*) specimen sampled from Southeastern Brazil. Values are presented as mg kg^-1^ wet weight.

| **Element** | **LOD** | **LOQ** |
| --- | --- | --- |
| Ag | 0,0003 | 0,001 |
| Al | 0,022 | 0,073 |
| As | 0,007 | 0,022 |
| Cd | 0,00017 | 0,00058 |
| Ce | 0,00021 | 0,00068 |
| Co | 0,0008 | 0,0027 |
| Cr | 0,009 | 0,028 |
| Cu | 0,008 | 0,027 |
| Dy | 0,00006 | 0,00019 |
| Er | 0,00005 | 0,00015 |
| Eu | 0,00005 | 0,00015 |
| Fe | 0,11 | 0,38 |
| Gd | 0,00028 | 0,00094 |
| Hg | 0,0024 | 0,008 |
| Ho | 0,000026 | 0,000085 |
| La | 0,0003 | 0,0011 |
| Lu | 0,00004 | 0,00014 |
| Mn | 0,0021 | 0,007 |
| Nd | 0,006 | 0,02 |
| Ni | 0,004 | 0,012 |
| Pb | 0,0016 | 0,0053 |
| Pr | 0,00007 | 0,00024 |
| Rb | 0,0005 | 0,0016 |
| Sm | 0,000005 | 0,000018 |
| Tb | 0,00004 | 0,00014 |
| Ti | 0,007 | 0,024 |
| Tm | 0,000021 | 0,000068 |
| V | 0,0006 | 0,0021 |
| Yb | 0,000003 | 0,00001 |
| Zn | 0,017 | 0,056 |

Table S2. Limits of Detection (LOD) and Limits of Quantification (LOQ) for each analyzed Polycyclic Aromatic Hydrocarbon determined in the thresher shark (*Alopias vulpinus*) specimen sampled from Southeastern Brazil. Values are presented as ng g^-1^ wet weight.

| **Compound** | **LOD*** | **LOQ**** |  |
| --- | --- | --- | --- |
|  |  |  |  |
| Naphthalene | 0.13 | 2.00 |  |
| Acenaphthylene | 0.13 | 2.00 |  |
| Acenaphthene | 0.13 | 2.00 |  |
| Fluorene | 0.27 | 2.00 |  |
| Dibenzothiophene | 0.27 | 2.00 |  |
| Phenanthrene | 0.27 | 2.00 |  |
| Anthracene | 0.27 | 2.00 |  |
| Fluoranthene | 0.27 | 2.00 |  |
| Pyrene | 0.27 | 2.00 |  |
| Perylene | 0.27 | 2.00 |  |
| Benz[a]anthracene | 0.13 | 2.00 |  |
| Chrysene | 0.27 | 2.00 |  |
| Benzo[b]fluoranthene | 0.13 | 2.00 |  |
| Benzo[k]fluoranthene | 0.13 | 2.00 |  |
| Benzo[a]pyrene | 0.27 | 2.00 |  |
| Benzo[e]pyrene | 0.10 | 2.00 |  |
| Indeno[1,2,3-cd]pyrene | 0.27 | 2.00 |  |
| Dibenzo[a,h]anthracene | 0.27 | 2.00 |  |
| Benzo[ghi]perylene | 0.27 | 2.00 |  |

Table S3. Limits of Detection (LOD) and Limits of Quantification (LOQ) for each analyzed Polycyclic Aromatic Hydrocarbon determined in thresher shark (*Alopias vulpinus)*. Values in ng g^-1^ wet weight.

| **Compound** | **LOD*** | **LOQ**** |
| --- | --- | --- |
|  |  |  |
| alpha-HCH | 0.056 | 5.26 |
| beta-HCH | 0.054 | 5.26 |
| gamma-HCH | 0.088 | 5.26 |
| delta-HCH | 0.056 | 5.26 |
| Aldrin | 0.103 | 5.26 |
| Isodrin | 0.029 | 5.26 |
| Dieldrin | 0.132 | 5.26 |
| Endrin | 0.018 | 5.26 |
| Heptachlor | 0.074 | 5.26 |
| Heptachlor epoxide A | 0.036 | 5.26 |
| Heptachlor epoxide B | 0.045 | 5.26 |
| Oxychlordane | 0.097 | 5.26 |
| gamma-Chlordane | 0.137 | 5.26 |
| alpha-Chlordane | 0.154 | 5.26 |
| o,p'-DDE | 0.130 | 5.26 |
| p,p'-DDE | 0.149 | 5.26 |
| o,p'-DDD | 0.058 | 5.26 |
| p,p'-DDD | 0.161 | 5.26 |
| o,p'-DDT | 0.172 | 5.26 |
| p,p'-DDT | 0.115 | 5.26 |
| Endosulfan I | 0.134 | 5.26 |
| Endosulfan II | 0.150 | 5.26 |
| Methoxychlor | 0.016 | 5.26 |
| Mirex | 0.132 | 5.26 |
